# Supplementary material for: Beer or Ethanol Effects on the Body Composition Response to High-Intensity Interval Training. The BEER-HIIT Study
Source: Nutrients. 2019 Apr 23;11(4):909. doi: 10.3390/nu11040909 (PMC6521009; doi:10.3390/nu11040909)
Supplement: Supplementary file 1 [file nutrients-11-00909-s001.pdf]

SUPPLEMENTARY MATERIAL

Table S1. Changes in body composition outcomes adjusted by baseline values (Model 1) adjusted by baseline values and sex (Model 2), by baseline values and age (Model 3).

		Analysis of covariance P value		
		F	P	η^2
Fat mass (kg)	Model 1	1.721	0.156	0.094
	Model 2	1.698	0.161	0.095
	Model 3	1.943	0.114	0.107
Waist Circumference	Model 1	**3.053**	**0.023**	**0.156**
	Model 2	**2.774**	**0.034**	**0.146**
	Model 3	**3.064**	**0.022**	**0.159**
Lean mass (kg)	Model 1	**6.683**	**≤0.001**	**0.288**
	Model 2	**6.604**	**≤0.001**	**0.289**
	Model 3	**6.283**	**≤0.001**	**0.279**
Fat mass (%)	Model 1	**3.660**	**0.009**	**0.182**
	Model 2	**3.459**	**0.013**	**0.175**
	Model 3	**3.697**	**0.009**	**0.185**
Visceral adipose tissue (g)	Model 1	0.652	0.627	0.038
	Model 2	0.503	0.733	0.030
	Model 3	0.688	0.603	0.041
Bone mineral density (g/cm^2)	Model 1	0.572	0.684	0.034
	Model 2	0.563	0.690	0.033
	Model 3	0.634	0.640	0.038
